# Supplementary material for: Spatiotemporal changes of eutrophication and heavy metal pollution in the inflow river system of Baiyangdian after the establishment of Xiongan New Area
Source: PeerJ. 2022 May 3;10:e13400. doi: 10.7717/peerj.13400 (PMC9074874; doi:10.7717/peerj.13400)
Supplement: Supplemental Information 1 [file peerj-10-13400-s001.docx]

| *WQI* of eutrophication | *WQI* of heavy metals | Description of water quality |
| --- | --- | --- |
| $WQI$≤0.8 | $WQI$≤1 | No pollution |
| 0.8<$WQI$≤1 | 1<$WQI$≤2 | Slight pollution |
| 1<$WQI$≤2 | 2<$WQI$≤3 | Moderate pollution |
| $WQI$≥2 | $WQI$>3 | Serious pollution |
