# Supplementary material for: Spatiotemporal changes of eutrophication and heavy metal pollution in the inflow river system of Baiyangdian after the establishment of Xiongan New Area
Source: PeerJ. 2022 May 3;10:e13400. doi: 10.7717/peerj.13400 (PMC9074874; doi:10.7717/peerj.13400)
Supplement: Supplemental Information 2 — Grade I: clean water from headwater and national conservation area that can be used for domestic purposes after simple disinfection, for recreational purposes and irrigation. II: fairly clean water that can be used as domestic water after treatment, for recreational purposes, for fish farming etc., and the area is strictly protected. III: water also can be used for domestic, recreational purposes after suitable treatment. IV: polluted water which can only be used as industrial water after treatment. V: heavily polluted water that should not be used at all. [file peerj-10-13400-s002.docx]

|  | Grade | | | | |
| --- | --- | --- | --- | --- | --- |
|  | Ⅰ | Ⅱ | Ⅲ | Ⅳ | Ⅴ |
| NH_4_^+^-N | 0.15 | 0.5 | 1.0 | 1.5 | 2.0 |
| TP | 0.02 | 0.1 | 0.2 | 0.3 | 0.4 |
| COD | 15 | 15 | 20 | 30 | 40 |
| Pb | 10 | 10 | 50 | 50 | 100 |
| Cd | 1 | 5 | 5 | 5 | 10 |
| Cr | 10 | 50 | 50 | 50 | 100 |
