# Supplementary material for: Spatiotemporal changes of eutrophication and heavy metal pollution in the inflow river system of Baiyangdian after the establishment of Xiongan New Area
Source: PeerJ. 2022 May 3;10:e13400. doi: 10.7717/peerj.13400 (PMC9074874; doi:10.7717/peerj.13400)
Supplement: Supplemental Information 3 [file peerj-10-13400-s003.docx]

|  | Dim.1 | Dim.2 |
| --- | --- | --- |
| Pb | 0.666 | -0.549 |
| Cd | 0.792 | -0.015 |
| Cr | 0.454 | 0.830 |
