# Supplementary figures and images for: Spatiotemporal changes of eutrophication and heavy metal pollution in the inflow river system of Baiyangdian after the establishment of Xiongan New Area
Source: PeerJ. 2022 May 3;10:e13400. doi: 10.7717/peerj.13400 (PMC9074874; doi:10.7717/peerj.13400)

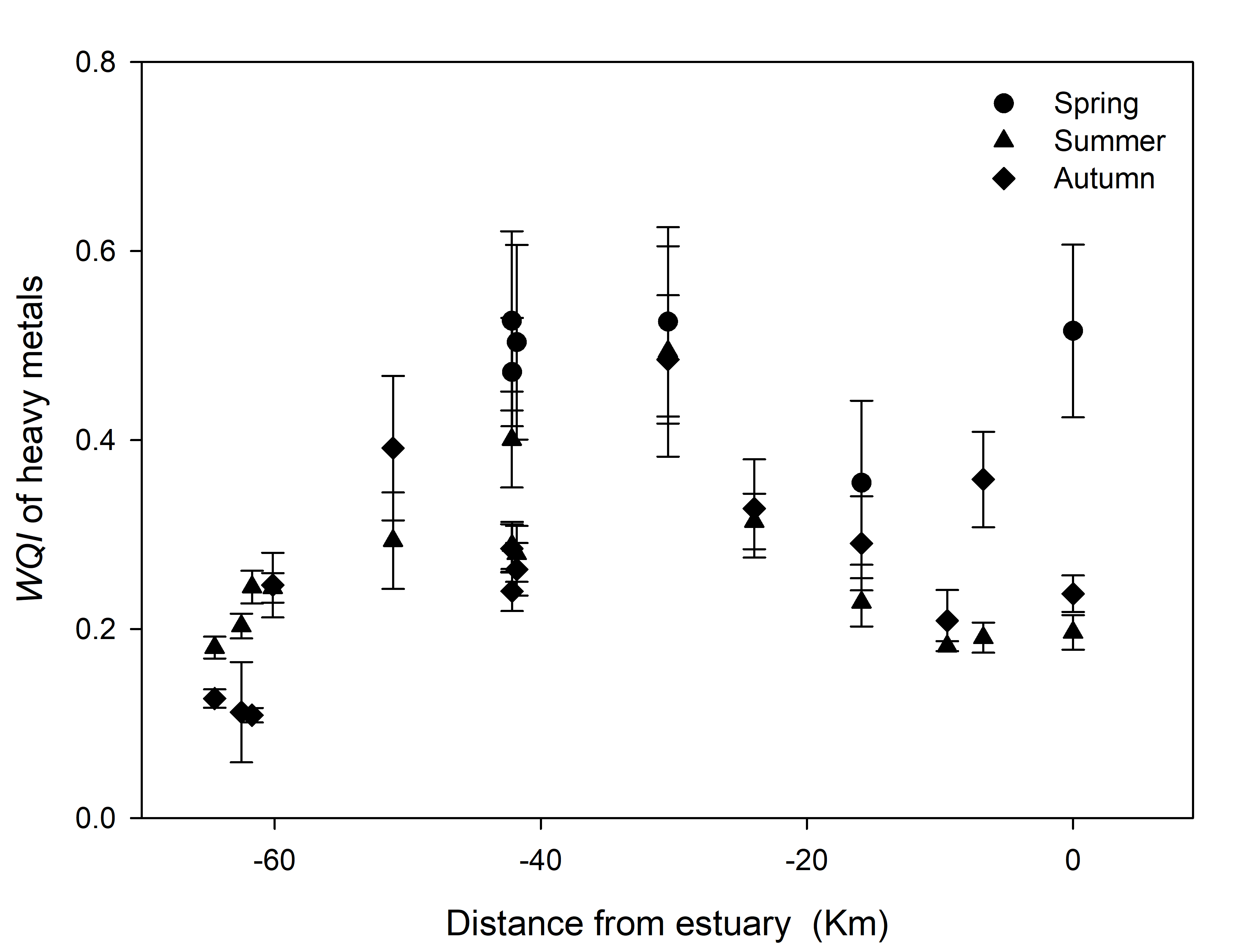

Supplement: Supplemental Information 4 [file peerj-10-13400-s004.png]
